# Feasibility and Reliability of Health-Related Physical Fitness Tests in Children and Adolescents with Hearing Impairment

**DOI:** 10.3390/children10020353

**Published:** 2023-02-10

**Authors:** Jiafu Huang, Jianmei Yang, Justin A. Haegele, Lijuan Wang, Sitong Chen, Chunxiao Li

**Affiliations:** 1 School of Physical Education & Sports Science, South China Normal University, Guangzhou 510006, China; 2 Adapted Physical Activity Laboratory, South China Normal University, Guangzhou 510006, China; 3 Department of Physical Education, Zhongshan Polytechnic, Zhongshan 528400, China; 4 Department of Human Movement Sciences, Old Dominion University, Norfolk, VA 23529, USA; 5 Center for Movement, Health, & Disability, Old Dominion University, Norfolk, VA 23529, USA; 6 School of Physical Education and Sport Training, Shanghai University of Sport, Shanghai 200438, China; 7 Institute for Health and Sport, Victoria University, Melbourne 8001, Australia

**Keywords:** repeatability, practicality, physical function, hard of hearing

## Abstract

Although research supports the feasibility and reliability of health-related physical fitness (HRPF) tests in typically developing children and adolescents, little is known regarding the feasibility and reliability of these tests for those with hearing impairments (HI). The aim of this study was to evaluate the feasibility and reliability of a HRPF test battery for children and adolescents with HI. A test–retest design with a one-week interval was conducted with 26 participants with HI (mean age: 12.7 ± 2.8 years; 9 male). The feasibility and reliability of seven field-based HRPF tests (i.e., body mass index, grip strength, standing long jump, vital capacity, long distance run, sit-and-reach, one leg stand) were evaluated. All the tests showed high feasibility (completion rate > 90%). Six tests indicated good to excellent test–retest reliability (all intraclass correlation coefficient [ICC] > 0.75) while the one leg stand test showed poor reliability (ICC = 0.36). Relatively large percentages of standard error of measurement (SEM%) and minimal detectable change (MDC%) were observed in the sit-and-reach test (SEM% = 52.4%, MDC% = 145.2%) and one leg stand test (SEM% = 107.9%, MDC% = 299.2%), whereas the rest of the tests demonstrated reasonable SEM% and MDC% values. Collectively, most of the tests can be feasibly and reliably used to assess HRPF for children and adolescents with HI.

## 1. Introduction

Hearing impairment (HI) refers to any hearing loss above 20 dB in either or both ears. HI can range from mild to profound in severity and may include those who are hard of hearing and deaf [1]. Globally, approximately 430 million people (34 million children and adolescents) or over 5% of the world’s population live with HI in 2021, and the number is expected to exceed 700 million by 2050 [2]. Second only to physical disability, HI is the second largest category of disability in China. Specifically, there are more than 22 million people with HI, of whom 581,000 are children and adolescents [3]. People with HI were reported to have poor physical and psychological performance and may face more health problems during growth and development [4,5].

Several studies have shown that children and adolescents with HI seem to have lower health-related physical fitness (HRPF) levels than their typically developing peers [6,7]. HRPF is one of two major components of physical fitness, along with skill-related physical fitness, and may include body composition, muscular strength, muscular endurance, cardiorespiratory fitness, and flexibility [8]. HRPF is recognized as an important predictor of health outcomes in all age groups, and physical fitness levels during childhood and adolescence can predict health status in adulthood [9,10,11]. Thus, improving the HRPF levels in childhood and adolescence is conducive to enhancing their health [11,12]. For example, children and adolescents with healthy cardiorespiratory fitness levels have a lower level of total and abdominal adiposity [12,13].

Low HRPF levels in children and adolescents with HI might be attributed to the lack of physical activity participation and prolonged sedentary behaviors in this group [14,15]. For example, research found that only 4% of deaf adolescents met the World Health Organization’s physical activity recommendation, and they showed higher levels of sedentary time than their peers without HI [14]. Low HRPF levels are concerning, as they are associated with high risks of a range of health-related outcomes, such as cardiovascular disease, diabetes mellitus, and poor mental health [16,17]. Statistics have indicated that about 17% of children and adolescents with HI suffer from depression and/or anxiety, and they are 2.4–5.3 times more likely to have these mental health problems than their hearing counterparts [18,19,20]. Therefore, intervention programs should be developed for the promotion of HRPF among children and adolescents with HI. 

HRPF assessment is an essential step for the development of intervention programs. The assessment outcomes (i.e., HRPF levels and conditions) can be used for exercise program planning, health promotion, and informing related research for the targeted group [21]. There are several well-established field-based HRPF tests for typically developing children and adolescents. For example, the Chinese National Student Physical Fitness Standard (CNSPFS) and FITNESSGRAM are commonly used assessments that have been developed and validated for assessing the physical fitness levels of children and adolescents without disabilities [22,23]. The CNSPFS consists of a wide range of tests (e.g., body composition, vital capacity, and sit-up) and is the official tool for assessing HRPF of children and adolescents without disabilities in China [22]. 

To date, the research on HRPF measurement has mainly focused on children and adolescents without disabilities [22,23], as well as those with intellectual and physical disabilities [24,25,26,27,28]. For example, the reliability, feasibility, and validity of HRPF test tools such as the Brockport Physical Fitness Test, SAMU-Disability Fitness Battery, and Assessing Levels of Physical Activity Fit test battery have been examined in disability groups, such as groups with intellectual disabilities and physical disabilities [26,27,28]. Interestingly, although a few studies have examined HRPF levels and correlates among individuals with HI [4,29], little research, to the best of our knowledge, has evaluated the feasibility and reliability of the HRPF tests in this group. Examining the feasibility and reliability of the HRPF tests, specifically for children and adolescents with HI, should be conducted to guide researchers and practitioners in test selection, refinement. and development. Therefore, the present study was undertaken to evaluate the feasibility and test–retest reliability of a HRPF test battery in Chinese children and adolescents with HI.

## 2. Materials and Methods

### 2.1. Study Design

To serve the purpose of the present study, a test–retest design with an interval of one week was conducted. 

### 2.2. Participants

Individuals were included if they met the following inclusion criteria: (1) children and adolescents (7–17 years); (2) diagnosed with HI of any level by the official competent administration (obtained from participants’ latest medical record); (3) able to perform physical activity without health risks; and (4) able to follow instructions to complete HRPF tests. Individuals were excluded if they (1) were below 7 or above 17 years of age; and (2) were diagnosed with a secondary disability condition in addition to HI (e.g., intellectual disability, physical disability, and visual impairment). Using PASS 15 (NCSS LLC., Kaysville, UT, USA), sample size calculation was conducted (i.e., power = 0.80, number of observations = 2, *p* = 0.05, effect size = p_0_: 0.70, p_1_: 0.90). The result showed that a minimum sample size of 19 participants was required for performing the test of intraclass correlation coefficient (ICC) [30]. Thus, our sample size was deemed adequate (see Section 3.1. Participants’ characteristics).

### 2.3. Measures

A panel of six experts consisting of researchers, special education teachers, and fitness instructors was formed. After two rounds of deliberation, the panel selected seven field-based tests to assess all five HRPF components: (1) body composition: body mass index (BMI); (2) muscular strength: grip strength and standing long jump; (3) cardiorespiratory fitness: vital capacity and long-distance run; (4) flexibility: sit-and-reach; and (5) balance: one leg stand. These tests were adopted from either CNSPFS or FITNESSGRAM [22,23]. The selection of these tests were mainly based on their simplicity, low cost, and participants’ communication styles owing to their HI [4,29].

#### 2.3.1. Body Composition

Height and weight were measured using an automatic height and weight scale (GMCS-IV, Jianmin, Beijing, China). Participants were barefoot and wore light clothes. Participants were asked to stand straight, with their feet flat on the instrument pedal and face straight ahead, with their hands rested on the sides of their body naturally. BMI was calculated as body weight in kg divided by squared height in m (i.e., kg/m^2^).

#### 2.3.2. Muscular Strength

The grip strength was used to measure the strength of hand and forearm muscles, especially of the finger flexors. The test was assessed using an electronic hand dynamometer (WCS-100, Xinman, Shanghai, China). Participants were asked to grip as hard as possible with their dominant hand after the test started. There were two trials with one hand, establishing 30 s of rest in between. The maximum score in kg out of the two trials was selected for analysis.

The standing long jump was selected to assess the explosive strength of lower extremities. Participants stood behind the jumping line with their feet naturally separated, and without their toes stepping on or crossing the line. Participants jumped as far as possible with both feet together by bending their knees and swinging the arms. The best of three trials was recorded in cm.

#### 2.3.3. Cardiorespiratory Fitness

Vital capacity was measured with an electronic spirometer (FHL-001, Jianmin, Beijing, China). Vital capacity refers to the maximum air volume that an individual can expel from the lungs after maximum inhalation. During the measurement, participants were instructed to completely enclose the turbine with their mouth, to inhale up to their maximum capacity, and then to exhale as maximum force as possible. Two trials were conducted with at least 15 s of rest in between and the maximum value in ml was selected for analysis.

The long-distance run is a field-based test for cardiorespiratory fitness and it includes two categories (female = 800 m; male = 1000 m). Participants were asked to run as fast as possible on a safe and flat field track for one trial. The total duration of the test, measured in min, was used for analysis.

#### 2.3.4. Balance

The one leg stand test was used to measure participants’ balance. During the test, participants were asked to stand with bare feet together, hands on hips, and eyes closed. The test started when participants lifted either foot and held it steady. The duration of steady state covered during the test was measured in s. Two trials were given, and the best performance was chosen for analysis.

#### 2.3.5. Flexibility

The sit-and-reach test was selected to measure participants’ flexibility. Participants sat in front of the measuring instrument and were on bare feet together and straight, with the soles of the feet touching the instrument board. Meanwhile, they were asked to bend the torso forward as far as possible and push the marker forward with their fingertips. Two trials were conducted and the best distance in cm was selected for analysis.

### 2.4. Procedures

This study was approved by the Ethics Committee of the South China Normal University on 1 July 2022 (SCNU-SPT-2022-038). Participants with HI were recruited from a public special education school for students with varying disabilities located in Southern China. The participants and their guardians were informed about the study objectives and agreed to participate in the study. Participants completed all the HRPF tests twice with a one-week interval. To ensure consistency in testing, both tests were conducted by the same research team at the same time and location (within the special school). The research team consisted of 10 researchers and research assistants who were familiar with conducting HRPF tests. Since the participants were diagnosed with HI, the tests were conducted with the assistance of sign language interpreters and task cards to ensure participants understood the test requirements. One test station was set for each of the tests (i.e., BMI, grip strength, standing long jump, one leg stand, sit-and-reach, vital capacity, and long-distance run). Participants completed the first six HRPF tests in a random order before they completed the long distance run. The long distance run was administered last as it was likely to cause excessive fatigue which could have affected participants’ performance on other tests. Participants were allowed to discontinue the tests if they felt uncomfortable or could not continue. Participants were not provided with any incentives for participation in this study.

### 2.5. Statistical Analysis

All the statistical analyses were performed using SPSS 25.0 (IBM Corp, Armonk, NY, USA). SigmaPlot 14.0 (Systat Software Inc., Palo Alto, CA, USA) was used to create the Bland–Altman plots. Descriptive statistics including mean (M), standard deviation (SD), frequency (*n*), and percentages (%) were used to describe participant characteristics. Feasibility was calculated based on completion rate of each test. Completion rates of >75%, 50–75% or <50% for a test was considered feasible, fairly feasible, or not feasible, respectively [24]. Additionally, chi-square tests were used to determine if completion rates were significantly different for sex and age groups. 

ICC tests (mixed two-way model, absolute agreement, single measures) with 95% confidence intervals (95% CI) were used to determine test–retest reliability, i.e., relative reliability. ICC values of >0.90 represent excellent reliability, 0.75–0.90 represent good reliability, 0.50–0.75 represent moderate reliability, and < 0.50 indicate poor reliability [31]. The standard error of measurement (SEM) and the minimal detectable change (MDC) were used to determine the absolute reliability: SEM = SD_baseline tes_t × √ (1 − ICC); MDC = SEM × 1.96 × √2 [24,32]. To allow the SEM and MDC to be independent of the units of measurement, the SEM% and MDC% were determined: SEM% = (SEM/M) × 100; MDC% = (MDC/M_both tests_) × 100. It is worthy to note that there were no well-established cutoffs for SEM% and MDC%. Finally, a Bland–Altman plot with 95% limits of agreement was constructed for visualizing the agreement between the two tests [33].

## 3. Results

### 3.1. Participants’ Characteristics

**Figure 1:**
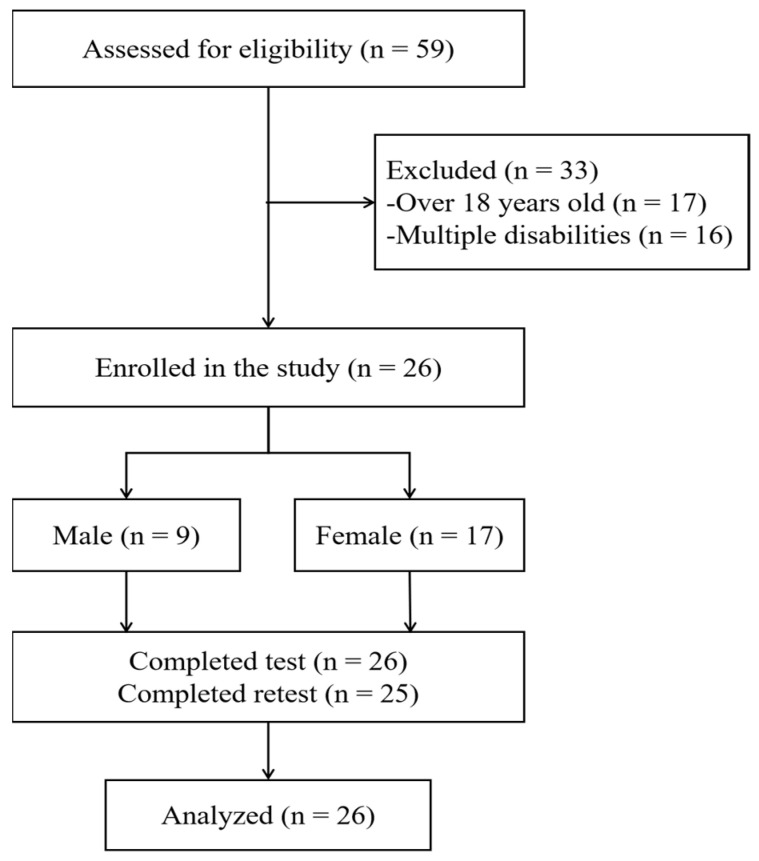
Flow chart of the selection of study participants.

As depicted in Figure 1, a total of 59 participants were assessed for eligibility and 33 participants were excluded. Of these, 16 were diagnosed with multiple disabilities, meaning that they were diagnosed with a secondary disability condition in addition to HI (e.g., intellectual disability, physical disability, and visual impairment), and 17 did not meet the age criteria (i.e., they were either below 7 years or above 17 years). Finally, 26 participants were included in this study; 9 of whom were male (M_age_ ± SD_age_: 11.9 ± 3.4 years) and 17 were female (M_age_ ± SD_age_: 13.1 ± 2.5 years). The majority of the participants were diagnosed with level I HI (80.8%), i.e., the most severe impairment level. In addition, there were no sex differences in age, height, and weight (all *p* > 0.05; see Table 1).

### 3.2. Feasibility

As shown in Table 2, all the HRPF tests were feasible for the participants with HI across the two test occasions and only one to two participants did not manage to complete the retest (test = 100%, retest > 92.3%). In addition, there was no significant sex or age difference in the completion rate of all the HRPF tests across the two test occasions (all *p* > 0.05).

### 3.3. Test–Retest Reliability

Table 2 shows descriptive statistics of all test scores, as well as the results of test–retest reliability (ICC, SEM%, MDC%). Good to excellent test–retest reliability was found for all the HRPF tests (ICC = 0.85 to 0.99), except for the one leg stand test, which showed poor test–retest reliability (ICC = 0.36). Regarding absolute reliability, there were relatively large SEM% and MDC% values for the sit-and-reach test (SEM% = 52.4%, MDC% = 145.2%) and the one leg stand test (SEM% = 107.9%, MDC% = 299.2%). The other tests generally showed reasonable absolute reliability values (SEM% = 1.6% to 12.9%, MDC% = 4.3% to 35.8%).

**Figure 2:**
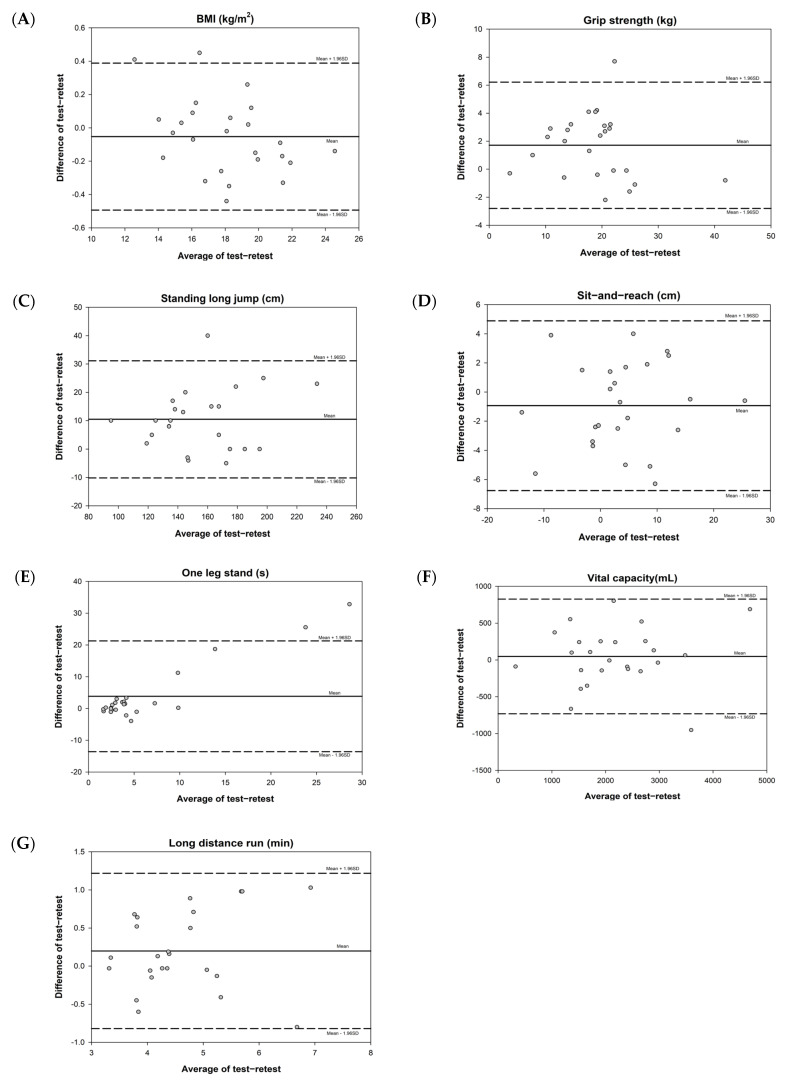
Bland–Altman plots of physical fitness tests. BMI = body mass index.

The Bland–Altman plots demonstrated no major systematic bias for all of the physical fitness tests excepted for the one leg stand test, for which the scores were mainly below the mean difference (see Figure 2). The 95% limits of agreement of physical fitness tests were as follows: BMI [−0.49, 0.39], grip strength [−2.81, 6.22], standing long jump [−10.17, 31.13], sit-and-reach [−6.76, 4.89], one leg stand [−13.59, 21.29], vital capacity [−731.54, 826.58], and long distance run [−0.82, 1.22]. Taken together, the majority of these tests appeared to have reasonable test–retest reliability.

## 4. Discussion

To the best of our best knowledge, this is the first study to examine the feasibility and reliability of HRPF tests in children and adolescents with HI. Our findings showed that the test battery consisting of seven HRPF tests showed a high level of feasibility. Meanwhile, all these tests produced good or excellent test–retest reliability scores (ICC ≥ 0.85) with the exception of the one leg stand test (ICC = 0.36). Furthermore, MDC% and SEM% values provided similar findings.

Based on our findings, many of HRPF test items can be recommended for use with children and adolescents with HI without much reservation. That is, the assessment of body composition using BMI, the assessment of strength using grip strength and standing long jump, and the assessment of cardiorespiratory fitness using vital capacity and the long-distance run, each of which performed well in feasibility and reliability tests in this study. This is perhaps not surprising, given that each of these tests have demonstrated good feasibility and reliability in other study groups [22,34]. For example, prior studies have demonstrated good feasibility and reliability for BMI among children without disabilities [35] as well as those with intellectual disabilities [27,28], whereas the grip strength test has been shown to have good reliability and feasibility for people with intellectual disabilities [27,28], bipolar disorder [36], and down syndrome [37]. The reliability coefficients of the standing long jump found in our sample (ICC = 0.89) are comparable with those in typically developing children [38], adolescents with an intellectual disability [21], and adults with bipolar disorder [36]. Regarding the assessment of cardiorespiratory fitness, the most common field-based fitness tests were the 20 m shuttle run test and the 6 min walk test [34]. However, we evaluated the cardiorespiratory fitness of participants with HI by borrowing the vital capacity and long-distance run tests from the CNSPFS in the present study. The decision was made on the basis of the local context, the characteristics of Chinese children and adolescents with HI, and the inputs from a panel of experts. Furthermore, several studies showed that the long distance run and vital capacity showed good feasibility and reliability for Chinese children and adolescents without disabilities [39,40] and these are parallel to our results (feasibility = 92.3–100%, ICC = 0.85–0.91). As such, researchers and practitioners using HRPF tests for children and adolescents with HI should feel confident in adopting these measures. 

Whereas many of the HRPF test items performed well in this study, issues emerged with regard to two test items. Firstly, the one leg stand test, which was used to measure balance ability, demonstrated weak reliability among our sample (ICC = 0.36, SEM% = 107.9%, MDC% = 299.2%). A recent study also found this test unreliable in people with intellectual disabilities [28]. Theoretically, balance is closely associated with the sensory system, particularly vision and hearing [41,42]. HI affects the development of balance due to damaged vestibular apparatus [43] and it could be difficult for people with HI to perform the one leg stand test with their eyes closed. Secondly, while the sit-and-reach test showed excellent feasibility (96.2%) and reliability (ICC = 0.94), which are consistent with previous findings in children and adolescents without HI [35], it produced relatively high SEM% and MDC% values (≥52.4%), suggesting the test results are unlikely to reflect “true” scores with our sample (i.e., this test has a relatively large measurement error). Because of this, the sit-and-reach test should be used with caution when assessing the flexibility of children and adolescents with HI. Indeed, several studies have shown that the sit-and-reach test was not a valid test to assess flexibility in adults, especially for lumbar extensibility [44]. Given these results, we would suggest that researchers and practitioners use caution when adopting the sit-and-reach test for children and adolescents with HI. We would also suggest that researchers and practitioners discard the one leg stand test and explore alternative tests to assess the balance of this target group.

The importance of HRPF in children and adolescents with HI suggests the need to develop systematic physical fitness assessments in health monitoring and promotion for this population [16,17,18,21]. Although some physical fitness test batteries, such as the Brockport Physical Fitness Test and Eurofit Special, have been designed for people with disabilities in western countries, the targeted groups of these batteries do not include people with HI [45,46]. For instance, the Brockport Physical Fitness Test is mainly used for measuring HRPF in children and adolescents with intellectual disabilities, visual impairment, cerebral palsy, a spinal cord injury, and physical disabilities [45]. Meanwhile, an official physical fitness test battery for people with disabilities (inclusive of HI) is currently not available in China. Thus, the findings of our study could shed light on the development of HRPF tests for people with HI. Regardless of this significance, there were two major limitations in the present study. First, our study merely focused on examining the feasibility and reliability of the seven HRPF tests. Other psychometric properties of these tests such as content validity, concurrent validity, and predictive validity should be examined in future research. In addition, the questionable test–retest reliability of the sit-and-reach test as well as the one leg stand test suggests the need to explore alternative HRPF tests in future. From the economic perspective, further studies could also explore alternative tests that will lead to fewer administrative burdens to participants and/or test administrators. Second, our sample was limited to children and adolescents with HI and the majority of them were diagnosed with level I HI, which makes it difficult to generalize the results to a larger group. Furthermore, our sample size together with participants’ age range (7–17 years) prevented us from conducting further analyses (e.g., age group difference in test–retest reliability). In future, it is necessary to recruit lager and more representative samples in terms of age, sex, and the severity of HI. 

## 5. Conclusions

The findings of this study provide some initial evidence on the feasibility and reliability of seven HRPF tests in Chinese children and adolescents with HI. Most of these tests (i.e., BMI, grip strength, standing long jump, vital capacity, and long distance run) show high feasibility and good to excellent test–retest reliability, and thus can be feasibly and reliably used for assessing HRPF in children and adolescents with HI. The one leg stand and sit-and-reach tests, however, should be used with caution. It is hoped that the findings of the present study could stimulate more research on HRPF in this understudied field.

## Figures and Tables

**Table 1:** Characteristics of the participants (*n* = 26).

	M ± SD or *n* (%)		
	Male (*n* = 9)	Female (*n* = 17)	Total (*n* = 26)
Age (years)	11.9 ± 3.4	13.1 ± 2.5	12.7 ± 2.8
Height (cm)	143.1 ±20.0	147.5 ±10.8	146.0 ± 14.4
Weight (kg)	36.7 ±15.9	40.7 ±9.2	39.3 ± 11.8
Age category			
7–12 years	6 (23.1%)	5 (19.2%)	11 (42.3%)
13–17 years	3 (11.5%)	12 (46.2%)	15 (57.7%)
Level of HI			
Level Ⅰ (≥90 dB HL)	6 (23.1%)	15 (57.7%)	21 (80.8%)
Level Ⅱ (81–90 dB HL)	0 (0%)	2 (7.7%)	2 (7.7%)
Level Ⅳ (41–60 dB HL)	3 (11.5%)	0 (0%)	3 (11.5%)

Notes: HI = hearing impairment. There are four severity levels of HI in China (Levels I to IV).

**Table 2:** Completion percentages and test–retest reliability in participants with hearing impairment (*n* = 26).

Test	Test Feasibility		Retest Feasibility		Test	Retest	ICC (95% CI)	SEM%	MDC%
	n	(%)	n	(%)	M (SD)	M (SD)			
BMI (kg/m^2^)	26	100	25	96.2	18.00 (2.90)	18.05 (2.82)	0.99 (0.99–1.00)	1.6	4.3
Grip strength (kg)	26	100	25	96.2	17.39 (7.70)	19.48 (7.22)	0.93 (0.73–0.98)	10.8	29.8
Standing long jump (cm)	26	100	25	96.2	147.92 (29.37)	158.92 (31.71)	0.89 (0.42–0.97)	6.7	18.4
Sit-and-reach (cm)	26	100	25	96.2	4.89 (8.96)	3.37 (8.89)	0.94 (0.87–0.97)	52.4	145.2
One leg stand (s)	26	100	25	96.2	4.12 (2.93)	8.07 (11.04)	0.36 (0.00–0.65)	107.9	299.2
Vital capacity (mL)	26	100	25	96.2	2135.88 (912.73)	2189.80 (967.08)	0.91 (0.82–0.96)	12.9	35.8
Long distance run (min)	26	100	24	92.3	4.54 (0.92)	4.70 (1.03)	0.85 (0.67–0.93)	8.2	22.7

Notes: BMI = body mass index; M = mean; SD = standard deviation; ICC = intraclass correlation coefficient; CI = confidence interval; %SEM = percentage of standard error of measurement; %MDC = percentage of minimal detectable change.

## Data Availability

Data are available from the corresponding author with the South China Normal University.
